# Replication of Overmolded Orthopedic Implants with a Functionalized Thin Layer of Biodegradable Polymer

**DOI:** 10.3390/polym10070707

**Published:** 2018-06-26

**Authors:** Ahmed Elkaseer, Tobias Mueller, Sabino Azcarate, Martin Philipp-Pichler, Thomas Wilfinger, Wolfgang Wittner, Manfred Prantl, Daniel Sampaio, Veit Hagenmeyer, Steffen Scholz

**Affiliations:** 1 Institute for Automation and Applied Informatics, Karlsruhe Institute of Technology, 76344 Eggenstein-Leopoldshafen, Germany; tobias.mueller2@kit.edu (T.M.); veit.hagenmeyer@kit.edu (V.H.); steffen.scholz@kit.edu (S.S.); 2 Faculty of Engineering, Port Said University, Port Said 42526, Egypt; 3 IK4-TEKNIKER, c/Iñaki Goenaga 5, 20600 Eibar, Spain; sabino.azcarate@tekniker.es; 4 Wittmann Battenfeld GmbH, 2542 Kottingbrunn, Austria; Martin.Philipp-Pichler@wittmann-group.com; 5 RHP Technology GmbH, 2444 Seibersdorf, Austria; thomas.wilfinger@rhp-technology.com; 6 Ernst Wittner Ges. m. b. H., A1140 Wien, Austria; wolfgang.wittner@wittner.at; 7 Alicona Imaging GmbH, 8074 Raaba, Austria; Manfred.Prantl@alicona.com; 8 Faculdade de Engenharia, Universidade Estadual Paulista (Unesp), Guaratinguetá 12516-410, Brazil; dsampaio@feg.unesp.br

**Keywords:** orthopedic implants, biodegradable polymer, functionalized surface, micro injection molding, process optimization

## Abstract

The present paper reports on the development of a biodegradable overmolded orthopedic implant: a metal bone fixing screw, which has been overmolded with a functionalized thin layer of biodegradable polymer to enhance cell adhesion during the healing process. The main challenges were to integrate precise, high-throughput and repeatable solutions to achieve a thin, defect-free structured polymer layer and to ensure a high and consistent implant quality. The work carried out entailed determining proper materials (Purasorb PDLG 5010) for the biodegradable overmolding layer and its economical substitute (NaKu PLA 100HF) to be used during initial tool and process development, designing the surface structure of the overmolded polymer layer, development of injection molding tools, as well as feeding and handling procedures. The injection overmolding process of Purasorb PDLG 5010 polymer was controlled, and the process parameters were optimized. In particular, the dominant process parameters for the overmolding, namely injection pressure, barrel temperature and mold temperature, were experimentally examined using a circumscribed three-factor central composite design and two quality marks; overmolding roughness and mass of polymer. The analysis of the experimental results shows that the mass of the overmolding is not feasible for use as the quality mark. However, the optimal parameters for the overmolding of a metallic implant screw with a thin, micro-structured polymer layer with a predefined roughness of the surface texture have been identified successfully.

## 1. Introduction

Mass production of miniaturized components still poses serious challenges to overcome in terms of precision manufacturing [1,2,3], but also offers great benefits in terms of production time and costs [4,5]. Micro injection molding (µIM) has shown enormous potential as a cost-effective technique to fabricate micro-components and/or meso-parts with intricate 3D features for various industrial applications [6,7]. This is particularly due to its ability to mass produce near-net-shape replication, molded from a wide range of engineering materials, with tight tolerance, high repetition quality and minimal material waste [8,9]. That is, µIM offers economical and ready-to-use products with high dimensional accuracy and high surface finish that ordinarily do not need further post-processing to improve the final quality [10].

Recently, µIM with its high capabilities has been considered a key enabling technology for numerous successful medical implementations, where implants and surgical instruments can be molded out of different biocompatible materials [11,12]. In particular, biodegradable polymers have received substantial attention as molding materials for medical applications [13] since, after a certain time, they can be absorbed and, e.g., can be used for drug delivery purposes [13,14,15]. Products made of such materials have been used for scaffold engineering [16], the fabrication of micro-needles [17], bone surgery and dentistry [18]. However, in addition to the high cost of such materials, products made of pure biodegradable materials are normally associated with weakness-inherited problems that preclude the commercial exploitation of such products [13,18]. Thus, multi-material parts, components made from different materials and assembled in a single part that cannot be dismantled into its components without destroying it, could be the answer to this problem. The reason for using different material components to manufacture one part, making its manufacturing process more complex, is to optimize the characteristics and tune the part’s properties to its functional requirements. The multi-material part offers an integration of properties, the sum of which is usually better than the properties of each material separately. The tunable properties of the multi-materials make them the perfect solution: light-weight, strong, wear-resistant, chemically inert, aesthetic, soft to the touch, hypoallergenic, heat and/or electricity conductor/insulator, etc. Almost every requirement can be met by the correct choice of the subcomponents of a part [19].

Orthopedic devices made solely from biodegradable polymers have been reported [20,21]. Furthermore, medical implants with functionalized surfaces for enhanced cell growth are currently being investigated by a variety of research groups [22,23,24]. Because biodegradable materials used for implants should pose as few complications as possible, rather costly materials are used [17,18]. Though, as previously stated, in addition to their high cost, products made of pure biodegradable materials are normally associated with weakness-inherited problems that hinder the exploitable use of such products. One possible solution to produce more affordably and robust medical parts is to make multi-material parts using mass manufacturing techniques such as injection molding. Especially, µIM also has extraordinary advantages in manufacturing multi-material parts. With this technique, light-weight, free-form, polymer parts with enhanced characteristics can be either overmolded or in-mold assembled into a different material [25,26]. Consequently, orthopedic devices can be manufactured as a bimaterial system, with metallic-based materials for the core that provide high strength at reasonable cost and that are overmolded by a functionalized thin layer of biodegradable polymer in order to stimulate cell growth and drug delivery.

To the best of the authors’ knowledge, there has been no implant screw made of a bimaterial system able to provide the strength for stable fracture fixation with minimal trauma to vascular supply and at the same time helping to create an improved environment for bone healing, which advantageously accelerates the patient’s return to previous mobility and function. Accordingly, the motivation for this research study is to meet and overcome the specific challenges in the fabrication of a functional µ-structured coating, made of biodegradable polymer, overmolded around an implantable medical metal headless compression screw.

**Figure 1:**
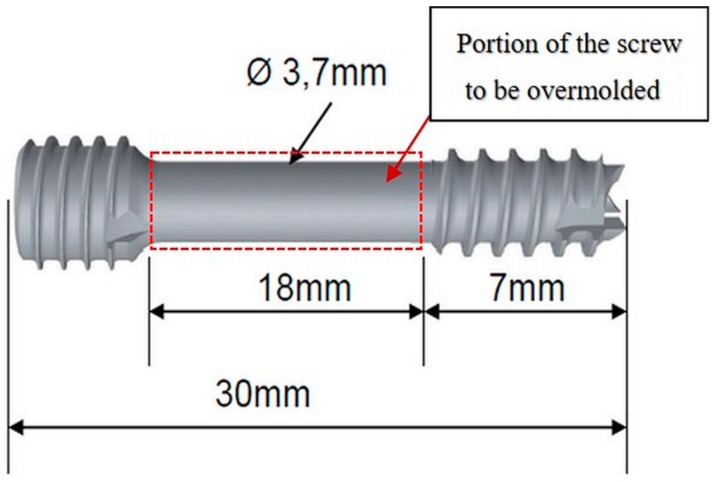
Headless compression screw and its dimensions.

The base part in this work is the headless compression screw shown in Figure 1. This is a self-drilling, self-tapping headless screw that allows surgeon-controlled compression combined with a simplified technique. The headless design helps minimize hardware prominence and soft tissue irritation. Currently, these screws are produced via regular machining processes such as milling, turning and polishing, but not with a biopolymer overmold. The challenge is to develop an implantable medical screw, with a functionalized biocompatible thin polymer surface layer with high accuracy and repeatable quality at acceptable cost.

The overmolding of biodegradable polymer will be over the smooth cylindrical portion of the screw (the 18-mm length between the two threaded areas; see Figure 1), previously manufactured by metal powder injection molding (MIM). This layer encompassing the cylindrical area will create an improved environment for bone healing due to the possibility of tunable drug dosing by controlling the biopolymer layer degradation rate, in which the drug is embedded and guiding cell migration and growth.

The bone screw implant requires special attention due to the high precision required in its manufacture, because the structure of the overmolded polymer layer, with its texture, is crucial for cell adhesion and growth, thereby reducing osseointegration time.

The aim of the present work is to develop a precise and high-throughput process chain, based on micro injection molding, and to optimize the overmolding process by identifying the optimum processing windows and process parameters, in order to guarantee the quality of the produced parts. This necessitates experimentally analyzing the overmolding process in order to understand the effects of the interactions of the independent parameters on the quality of the polymer overmolding. However, the development of the experimental setup that is comprised of the overmolding tool, handling and feeding system and the characterizing instrumentation is also addressed.

## 2. Experimental Work

In this section, the experimental work is presented. In particular, the selection of the biodegradable material and the rationale behind this determination is discussed followed by an outline of the design of the polymer layer to be overmolded. Then, the development of the injection molding setup is given. After that, the injection molding process and the design of the experiment to carry out the experimental trials are detailed. Finally, the characterization process is explained.

### 2.1. Material Selection

The metal core has to be made of a medical-grade material. Materials that fulfil these requirements are titanium and/or 316 L stainless steel. In this study, 316 L stainless steel is used to manufacture the core of the medical implant. This is because, in addition to its sterilization ability and being approved by the FDA, 316 L stainless steel is a cost-effective material with superior properties of high tensile strength, good formability and high corrosion resistance [27,28,29,30].

The thin layer to be molded over the metal core has three main tasks: drug delivery, enhancing cell growth and degradability. The requirements for such a material are shown in Table 1.

There is a variety of materials available on the market for the overmolding, of which four different types were considered:

- PGA (poly(glycolic acid); Purasorb PG S; Corbion Purac);
- PLGA (poly(lactide-co-glycolic acid); Purasorb PLG 8553; Corbion Purac);
- PDL (poly-d-lysine; Purasorb PDL 02A; Corbion Purac);
- PDLG (copolymer of dl-lactide and glycolide; Purasorb PDLG 5010; Corbion Purac).

All of these fulfil the requirements listed above except the degradation time of 28 days. This generally is the key factor when evaluating possible biodegradable materials since only a few of those that are commercially available degrade in such a short time. Purasorb PDLG 5010 from Corbion Purac was chosen as the most suitable material as its degradation time ranges from four to six weeks.

**Figure 2:**
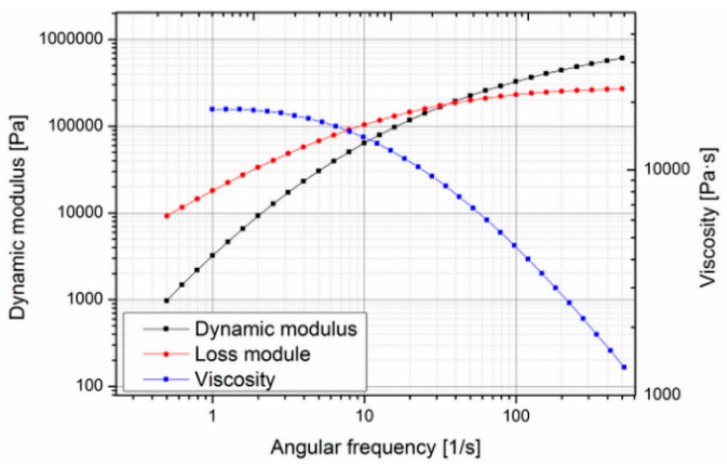
Rheological behavior of Purasorb copolymer of d-lysine-lactide and glycolide PDLG 5010 at 140 °C.

**Figure 3:**
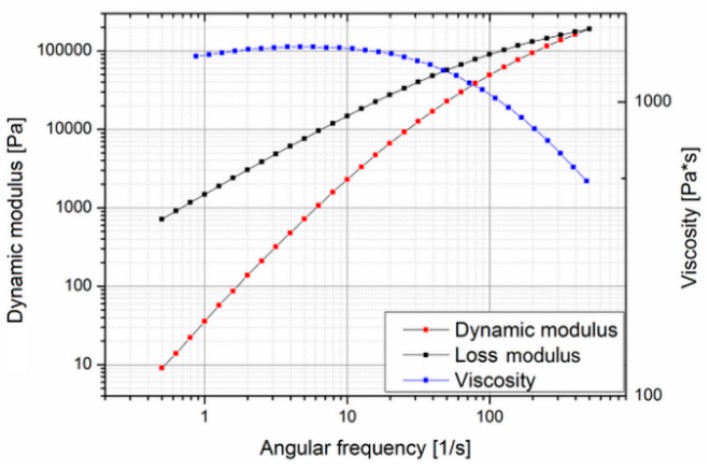
Rheological behavior of Purasorb PDLG 5010 at 160 °C.

Since this material is very expensive (about 3000 €/kg), it was decided to search for a suitable substitute material for the preliminary molding trials, during the tool development phase, and switch to the actual biodegradable material for the design of experiment and optimization study reported in this paper. In order to find a less expensive replacement, the actual properties essential for the molding process such as rheological behavior have to be known. In the case of Purasorb PDLG 5010, little data were available: density, drying conditions, mold temperature, melt temperature and molecular weight [32]. Since no data on the rheological behavior were available in the literature, the material was characterized using a cone-plate-rheometer. Measurements were carried out at different temperatures to determine the zero viscosity (Figure 2 and Figure 3).

**Figure 4:**
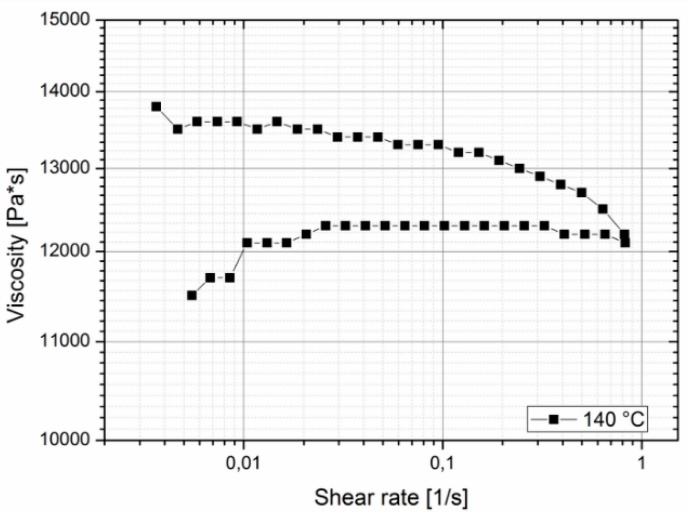
Shear rate vs. viscosity diagram showing the thixotropic behavior of Purasorb PDLG 5010.

The zero viscosity shown at 140 °C is 18.60 × 103 Pa·s, and at 160 °C, it was evaluated as 1.54 × 103 Pa·s. The measurements also showed that the viscosity of Purasorb PDLG 5010 changes with time, so a thixotropic behavior of the material cannot be precluded. This was determined by carrying out a cycle from low to high shear rates and back again at 140 °C. In Figure 4, a hysteresis effect, which is commonly associated with thixotropic materials, can be seen; the curve measured with increasing shear does not coincide with the curve measured with decreasing shear.

**Figure 5:**
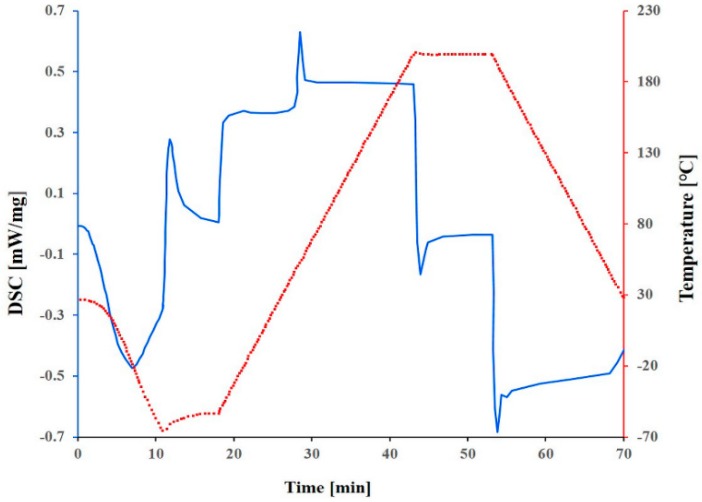
Thermogram of Purasorb PDLG 5010.

**Figure 6:**
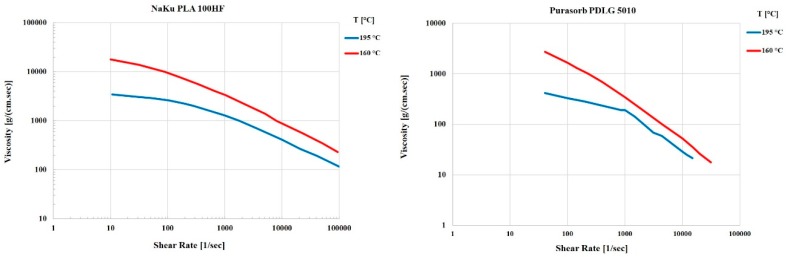
Comparison of the rheological behavior of NaKu PLA 100HF (left) and Purasorb PDLG 5010 (right).

To get more information on the thermal behavior of Purasorb PDLG 5010, the evaluation of the thermal transition of Purasorb PDLG 5010 was made using differential scanning calorimetry (DSC). The results are shown in Figure 5. The value of the specific heat capacity of PDLG 5010 was determined (1.97 J/g °C) similar to the specific heat capacity value of polylactide (2.16 J/g °C) [33]. Thus, and based on the results of the rheological and thermal measurements for the Purasorb PDLG 5010 materials being considered for the polymer overmolding, the costly biodegradable material was replaced with a polylactide (PLA) from NaKu (NaKu PLA 100HF) during the tool development phase, which has a relatively similar viscosity and rheological behavior; see Figure 6. Note that NaKu PLA 100HF was only used during the development and re-engineering of the experimental setup. It is worth emphasizing that the processing of Purasorb PDLG 5010 was largely determined by the same parameters as investigated in this stage. However, this enables the expensive actual material to be used only for the design of experiment and optimization study presented in this paper.

### 2.2. Feature Design of the Overmolding Polymer Layer

The biodegradable polymer overmolding the metallic implant screws is structured with a surface pattern advantageous for the settlement of new bone cells. With this, it is possible to increase cell growth, and any gap generated by the implantation of the screw can be reduced faster, resulting in more rapid stability. Furthermore, drugs that support the growing of new bone cells can be incorporated in the polymer, as well. While degrading, the polymer will release the drug in a controlled way.

**Figure 7:**
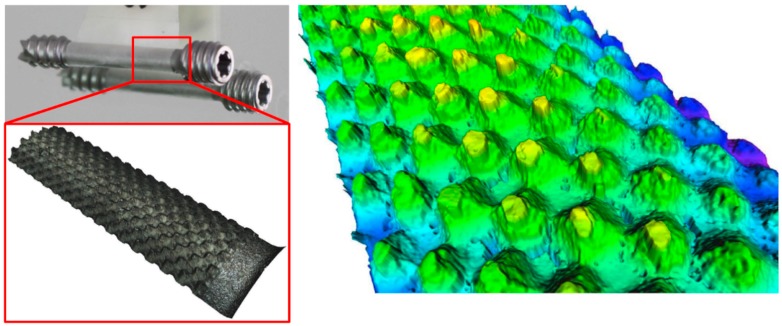
Surface structure for overmolded polymer; overview of the structure (**left**) and detailed measurement of the structure (**right**).

The patterning of the thin polymer layer was adapted to the injection molding process and is based on previous research carried out on the boosting of cell growth [34,35]. In particular, for better adoption of the implant by bone cells, the structure of the surface should have pores or indentations with lateral and vertical dimensions in the range of 100–400 μm. The chosen structure consisted of a right frustum of a square pyramid (see Figure 7). These shapes provided a good foundation in which the bone cells could grow and get a firm footing on the implant. Furthermore, these pyramids can be easily modified and provide a simple and reliable mold design. In the area of the parting line of the mold, the structure was flattened to allow for easy opening without deformation of the polymer surface. However, the structured area is sufficient to allow testing for the growth of the human cells. The final overmolding has a layer thickness of 50 µm in the thinner areas; the frustums are positioned in a continuous regular pattern with a spacing of 150 µm and feature a base width of 150 µm and a height of 120 µm; see Figure 7. In order to quantitatively describe the designated structure, an equivalent roughness average parameter (Ra) was utilized. According to the predefined specifications, a value of 40 µm Ra was targeted, as it is equivalent to the pattern dimensions of the overmolding layer mentioned above.

This selective texturing design is shown in Figure 6, which when applied to the external surface of the biopolymer layer, favorably accelerates the patient’s return to previous mobility and function. The bone screw implant requires high precision manufacturing since the structuring of the overmolded polymer layer is crucial for cell adhesion and growth, thereby reducing the osseointegration time.

It is worth emphasizing that the micro-structure of the surface of the metallic-based screw was modified with an analogical structure, as has been established on the polymer. This enables PDLG 5010 to create an interface between the polymer and the metal phases to lock up the polymer in its position and to improve the adhesion of the polymer layer to the metal screw.

### 2.3. Tool Development

For the production of the bone screw implant, two separate injecting molding tools were manufactured. One tool for the metal screw component made via MIM and one for the polymer overmolding. The tool for the metal component featured a cavity representing the finished product enlarged by a scaling factor, which was determined by the feedstock and is needed to compensate for the shrinkage of the part during the sintering process. However, as there is no novelty in producing the implant metal screw, the present paper reports only on the overmolding process of the biodegradable polymer layer.

**Figure 8:**
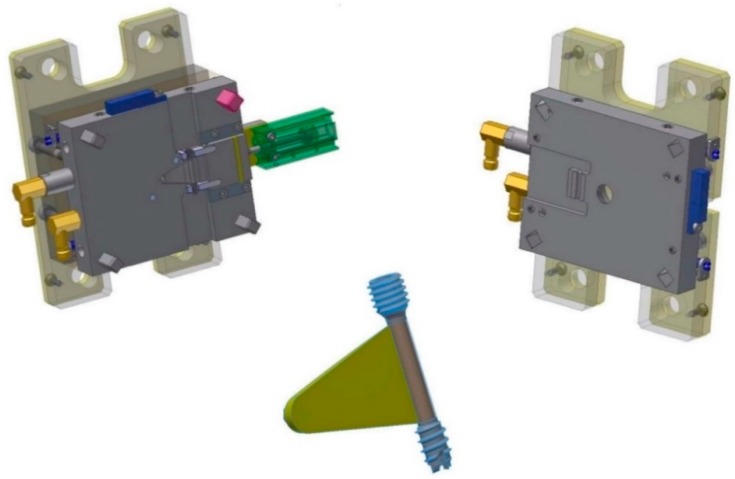
3D CAD models of the two parts tool for the polymer overmolding and the overmolded screw.

**Figure 9:**
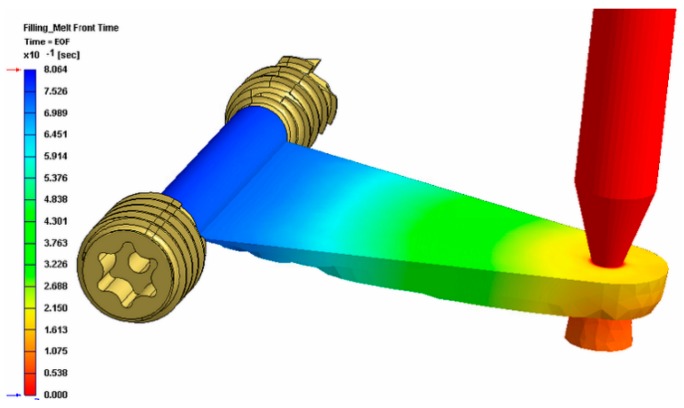
Simulation result of the overmolding cycle.

Figure 8 shows the tool for the overmolding process, while Figure 9 illustrates a simulation result of the overmolding cycle conducted using Autodesk Moldflow^®^. The developed tool features a thin film runner and gating system that expands over the whole middle section of the screw body to ensure a fast and homogenous filling of the cavity for the overmolding process. For overmolding of the metal screw, the part is placed inside the mold. As the wall thickness of the area to be overmolded is very low (~200 μm), and furthermore there is a structured surface to be filled, the film gate option (Figure 8) was chosen to reduce the necessary flow length as much as possible. The projected structure will impose many flow restrictions that will further decrease the attainable maximum flow length.

The simulation results of the injection cycle showed that a complete filling of the very thin overmolding area should be possible with the designated material within 0.8 s (see Figure 9). The tool also features an interchangeable cavity with exchangeable inserts for clamping the metal screw during the injection of the polymer on the nozzle side.

The screw is overmolded only in the region between the two threads (see Figure 1), where the polymer encases the whole shaft and cannot be removed without destroying the overmolding; nor can the coating be stripped in the axial direction, as there is a stepped zone in the screw that prevents axial movement of the polymer coating.

**Figure 10:**
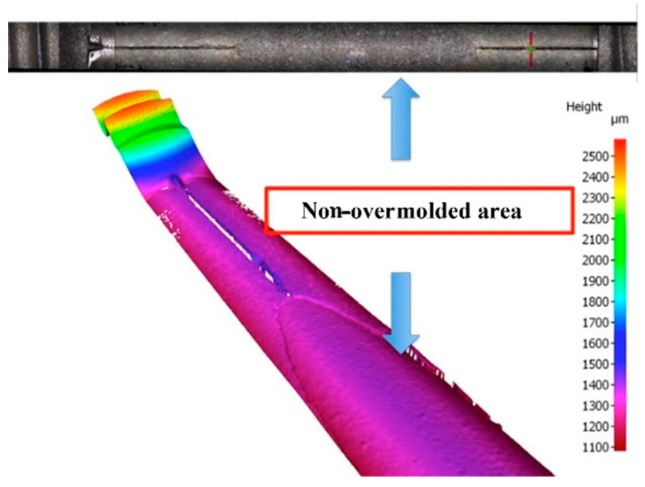
Picture (**top**) and surface scan (**bottom**) of the non-overmolded area due to bending of the metal screw.

**Figure 11:**
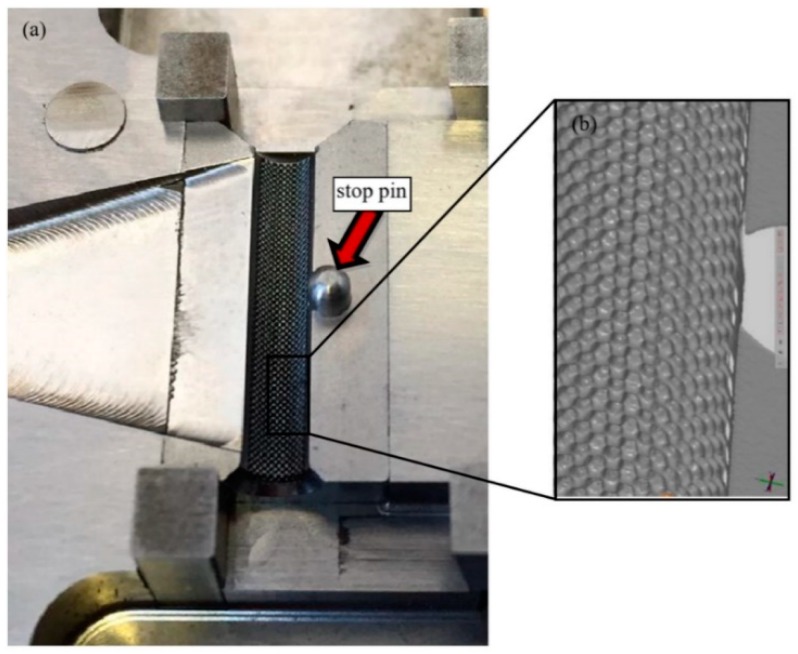
Final tool for overmolding (**a**) with additional stop pin to prevent bending of the metal screw insert and (**b**) 3D scan of the surface details.

However, the tool for the polymer overmolding had to be re-engineered and adapted after initial trials. The initial tests showed that the force of the injected polymer material caused a bending of the metal screw, which hindered the complete filling of the overmolding volume because the metal insert came in contact with the cavity walls (Figure 10). FEM simulations showed that the problem could not be solved by using a different material for the bone screw (e.g., titanium), and therefore, the tool was modified by adding an additional pin that prevented the metal insert from being deformed and thus ensured the overmolding of a maximum of the designated area (Figure 11a). Figure 11b shows a 3D scan of the surface pattern structure that was produced by micro-milling.

### 2.4. Feeding and Handling Process

**Figure 12:**
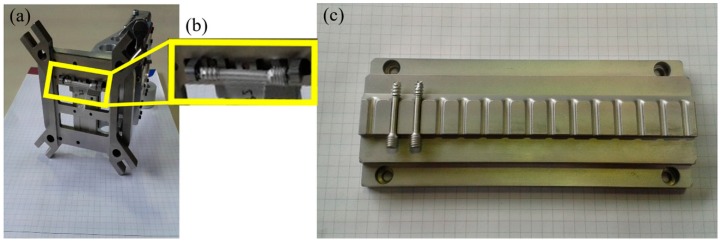
Equipment for the automation process (**a**) and (**b**) automation gripper and (**c**) tray unit.

The first step was feeding the implant screws into the injection molding machine. They come in trays, already sorted from the previous MIM process, and are placed within the workspace of the robot. To feed the metal screw into the overmolding tool, a gripper unit and a tray unit were used to develop a fully-automated process for mass production of the bone screw implant. A gripper has been developed, to be mounted on the robot, which will place the screw in the mold and then, later, remove it. Both components are shown in Figure 12.

**Figure 13:**
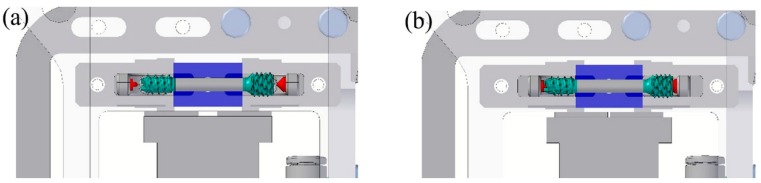
Screw gripper (red bits) (**a**) opened and (**b**) closed.

**Figure 14:**
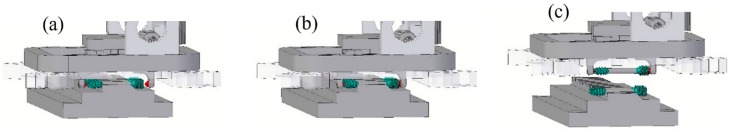
Screw gripping process from the tray unit (**a**) approaching; (**b**) holding and (**c**) moving to feed the overmolding tool.

The way the gripper holds the screw is shown in Figure 13. The goal is not to damage the thread or the overmolded polymer, and therefore, the gripper acts on the ends of the screw as shown in Figure 13a,b. Figure 14 shows the process of gripping the screw from the tray.

The final step is divided into three parts. First, the gripper is brought by the robot to the front of the mold with the mold brackets open. Then, the gripper moves to the cavity guided by square leader pins, extracts the screw from the storage unit and takes it to the mold for the injection process. The mold brackets close, and the gripper opens. The robot removes the gripper with the screw remaining in the mold.

In the next stage in the sequence, the rotary table moves to the injection side, and the molding process starts. After the molding is complete, the rotary table moves to the ejection side, and the gripper grips the screw and deposits it in its defined position in the tray. After that, the robot picks up the next screw to insert it into the mold.

### 2.5. Injection Molding Process

Manufacturing of the metal screws was performed at RHP Technology’s facilities in Seibersdorf, Austria, using a Battenfeld HM400/60 40 injection molding machine with a closing force of 40 tons. The material used for the screw was a 316 L feedstock with RHP’s own binder system. The parts were solvent debonded in a LÖMI EDA-50 solvent debinding furnace. Debinding was conducted at 40 °C for 8 h with subsequent drying under vacuum for three hours. After solvent debinding, the screws were put on specially-designed alumina setters for sintering. The special design was necessary to avoid warpage, which would render the screw unusable. Sintering took place in an Enlink Series 3002T MIM furnace. During the sintering process, a hydrogen atmosphere at a pressure of approximately 300 mbar was applied. Since the metal powder was fine (5 µm), sintering took place at a temperature of 1050 °C.

**Figure 15:**
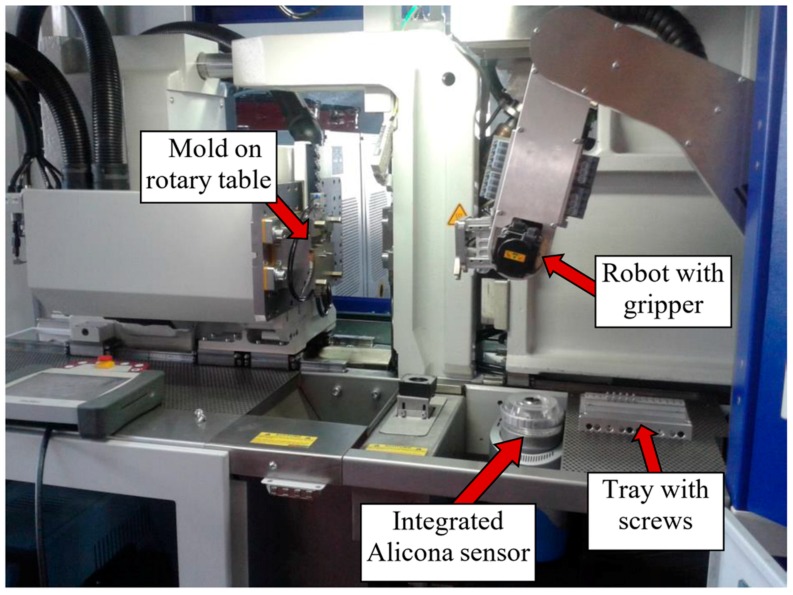
Processing setup: mold on rotary table, integrated Alicona sensor, tray with screws prepared for inserting and overmolding and robot with gripper.

The replication trials for the overmolding of the polymer material were carried out at the facilities of Wittmann Battenfeld GmbH (Kottingbrunn, Austria) on a Micropower 15/10-ton injection molding machine with a plunger diameter of 8 mm. The full setup is shown in Figure 15.

As mentioned earlier, during the development and re-engineering of the final working setup, PLA NaKu 100 as an economic substitute polymer was used to carry out the initial segments of the experimental trials. However, the design of experiment study and consequently the experimental trials to optimize the overmolding process were conducted using the biodegradable polymer, Purasorb PDLG 5010.

### 2.6. Design of the Overmolding Process Experiment

A design of experiment (DoE) study was carried out to determine the influence of the different process parameters, the interaction effect of the independent parameters on the quality of the polymer overmolding and their optimization. The selected design was a circumscribed three-factor central composite design that required five levels for each independent parameter, and the number of trials was reduced by applying a reduced factorial design to the experiments.

The parameters chosen for the experimental trials were injection pressure, barrel temperature and mold temperature, which are known to be the main influences in the process [36]. As already mentioned, roughness and mass values were used as quality marks. Since five levels are required for each parameter, the DoE experimental plan resulted in a total of 22 trials and 15 different parameter sets (there were eight trials, 10–17 inclusive, with the parameter set index at the central point). Table 2 shows for each trial number the parameter set index and the corresponding parameter values.

After set up and stabilization, five parts were produced for evaluation purposes for each trial number, resulting in 110 runs. The molding parameters were continuously measured with a PSP data acquisition system throughout the whole process. An average of the quality marks was obtained for each trial number. To analyze the data, MATLAB and Microsoft Excel were utilized. If sufficient information can be generated from the acquired data, a direct in-line quality control can be achieved by monitoring the most influential process parameters and thereby adapting the production process even while it is in operation to counteract changing manufacturing conditions within the automated process.

### 2.7. Characterization of Overmolded Parts

**Figure 16:**
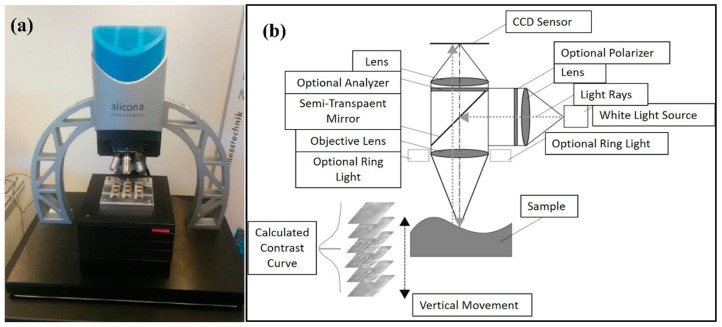
(**a**) Alicona microscopic assessment of implants and (**b**) principle of focus variation.

In order to check the quality of the produced parts, measurements of the structured area were carried out using an Alicona InfiniteFocus G5 system (Figure 16a) equipped with a 10× lens, which features a resolution of 1.76 µm in the lateral direction and 0.1 µm in the vertical direction. The Alicona InfiniteFocus G5 system exploits the so-called focus variation technique to gather topographical data from the variation of focus, as depicted in Figure 16b. In particular, with the aid of the semi-transparent mirror, the emitted light rays are injected through the optical path system and focused onto the surface of the sample to be examined. Then, the light rays are reflected into altered directions according to the variation of specimen topography, hit by the light, where these light rays are gathered by the light-sensitive sensor behind the semi-transparent mirror. It is worth stating that only small portions of the object are sharply captured, equivalent to the working depth of field of the optics. However, the optic system is continuously shifted along the vertical axis while uninterruptedly imaging the topography of the surface.

In this study, the measuring system is used to detect and measure flaws such as non-overmolded areas or incomplete filling of the pyramidal structures. The Alicona special algorithm for inspecting the screw was used to calculate the roughness of an area over a defined circumference of the screw. Thus, calculation times were massively reduced. In addition to measuring the bone screws, the Alicona InfiniteFocus R25 sensor system was also integrated directly into the injection molding machine to carry out similar measurements directly during the process, as shown in Figure 15.

The mass of the polymer was determined by subtracting the measured mass of the metal screw insert prior to overmolding, from the measured mass of the whole part after the replication step. This was done using a high precision laboratory scale.

The roughness (texture) values and the mass of the polymer will be used as quality marks in the analysis with higher values of both representing better part quality.

## 3. Results and Discussion

**Figure 17:**
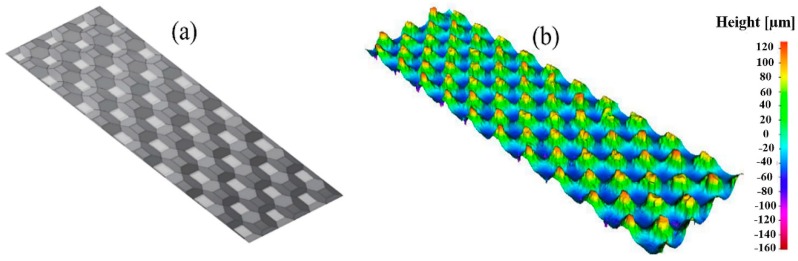
Surface structuring of polymer overmolding: (**a**) CAD model and (**b**) measurement result.

The calculated surface roughness of the implant is a good indicator of the quality of the bone implant interface. Parts with an adequate level of surface roughness can be described as acceptable. The structures were evaluated by determining the arithmetic average of the absolute roughness values (Ra) (Figure 17), which can be directly correlated with the height of the pyramidal structuring. Therefore, this value was chosen as the first quality mark for further process analysis. The Alicona system can present the 3D geometry in a way that helps to see differences more easily, as displayed in Figure 16.

**Figure 18:**
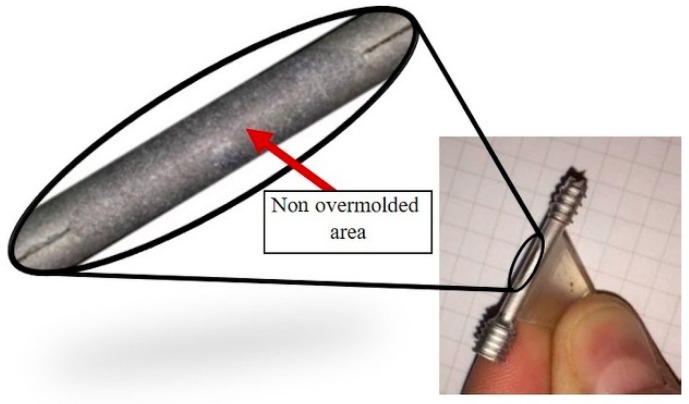
Screw showing non-overmolded area.

**Figure 19:**
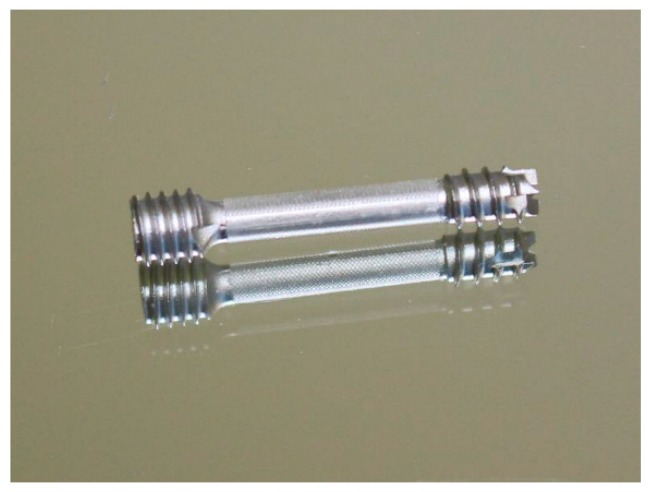
Overmolded screw.

**Figure 20:**
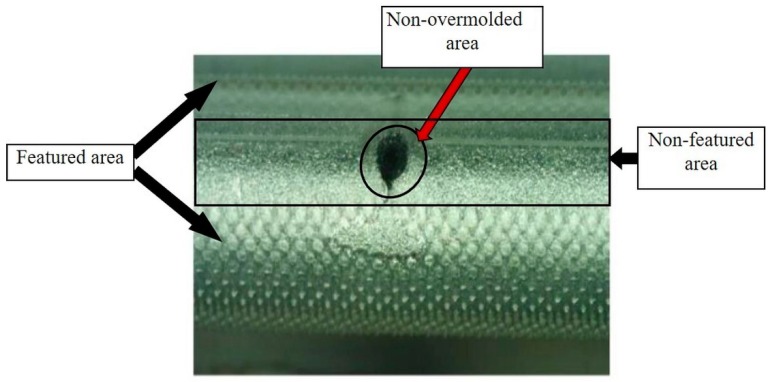
Mold with support for the screw.

As previously stated in Section 2.3, after the initial overmolding trials, the screws showed an area where no overmolding occurred (see Figure 18). The injection pressure necessary to fill the gap of just 200 microns was necessarily high and generated substantial bending forces on the screw during injection of the polymer. The necessary hole for the guiding wire required during implantation of the screw weakened the wall of the screw; the applied pressure overcame material resistance and the screw bent. However, after re-engineering the tool using the stop pin shown in Figure 10, the second molding trials were performed, and the overmolded parts were of acceptable quality. Figure 19 shows an implant screw with the central portion covered by the biodegradable polymer. Figure 19 shows the area where the screw was in contact with the support pin. The presence of the pin means there is a not overmolded area on the screw, where the polymer is non-existent (Figure 20), but this is very small and does not affect the functional performance of the implant screw, as it is located in the non-featured area.

However, due to the transparency of the used polymer to the light of the Alicona InfiniteFocus R25 sensor system that had been directly integrated into the injection molding machine, no direct in-line measurements were possible. To make the surface structures visible to the system, the parts have to be coated with reflective nanoparticles. Of course, items coated with nanoparticles that have not been medically assessed as fulfilling the requirements for medical implants cannot be used for in-line inspection. However, this technique is quite acceptable for off-line inspection of specimen that will be eliminated from the regular product, as they do not fulfil the requirements for medical implants any more. It is worth stating that this is not the case for ALICONA Infinite Focus G5 system, shown in Figure 16, which has the ability to address such a lighting issue. Consequently, the overmolded parts were assessed off-line using the Alicona InfiniteFocus G5 system, as shown in Figure 16.

**Figure 21:**
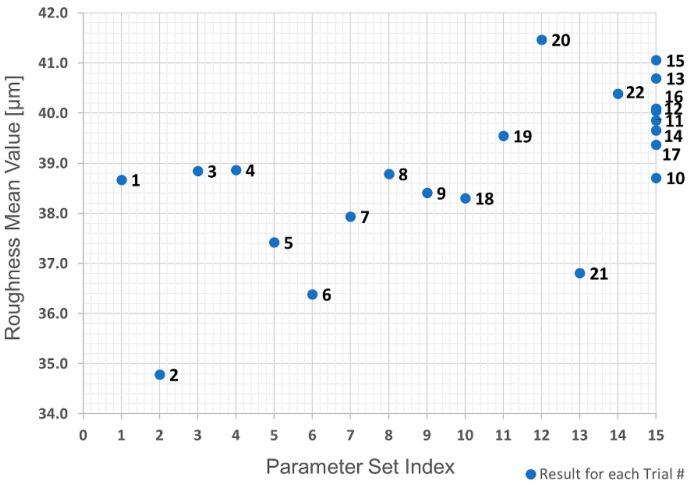
Replicate plot: average roughness for each trial number.

**Figure 22:**
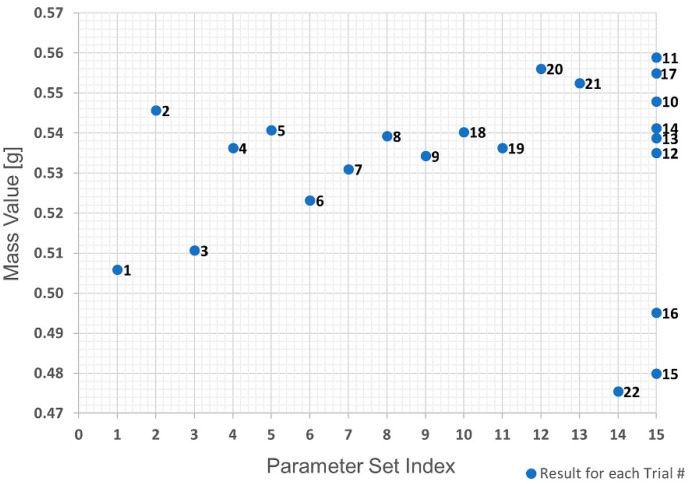
Replicate plot: average mass value for each trial number

The first analysis was the evaluation of the raw data obtained in the trials using a replicate plot for roughness and average polymer mass value, as shown in Figure 21 and Figure 22. Since the variation of average roughness for the eight trials with the replicated parameter set shown as Index 15 in Table 2 is much smaller than the variation over the entire investigation series (see Figure 21), it was concluded that the replicate error would not complicate the data analysis, which means a good model could be obtained. This is not the case with the polymer mass value. Figure 22 shows that the variation in the replicated parameter set is almost the same as the variation across the entire investigation. Based on these results, the polymer mass value quality mark was discarded due to the infeasibility of obtaining a respective adequate regression model.

Linear regression was used to fit the data and obtain a process model. Initial efforts were attempts to model using only linear terms for the interactions between each predictor, but the results were not acceptable with R² equal to 0.567 and a *p*-value equal to 0.0292. However, using squared terms for the predictors in the model gave a value of R² = 0.921 and a *p*-value = 0.0000254, which can be considered very good results.

Analysis of variance (ANOVA) for the roughness obtained is presented in Table 3. Looking at the *p*-value, one can conclude that the four most significant terms are, in order, (i) interaction between injection pressure and mold temperature; (ii) (injection pressure)²; (iii) interaction between barrel temperature and mold temperature; and (iv) barrel temperature.

**Figure 23:**
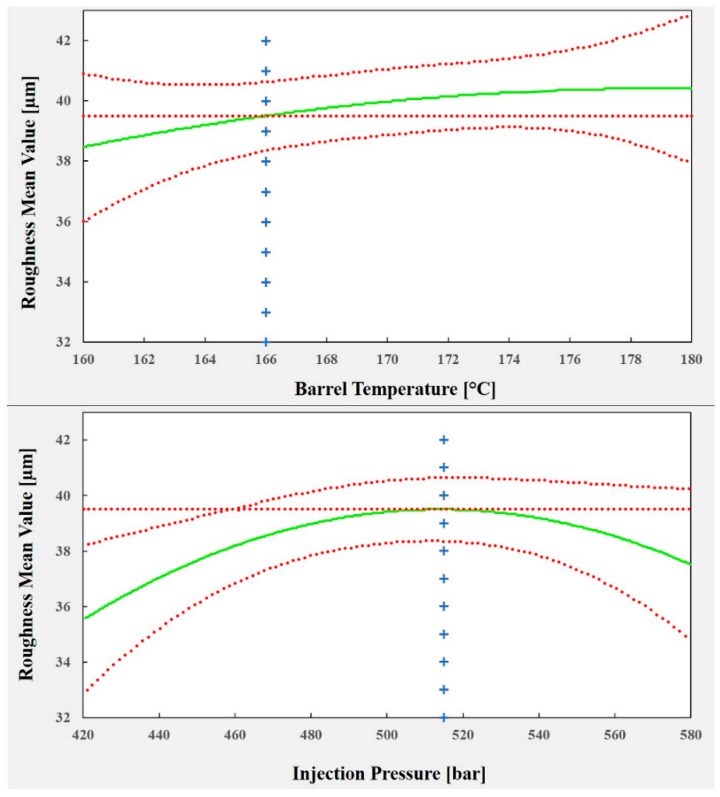

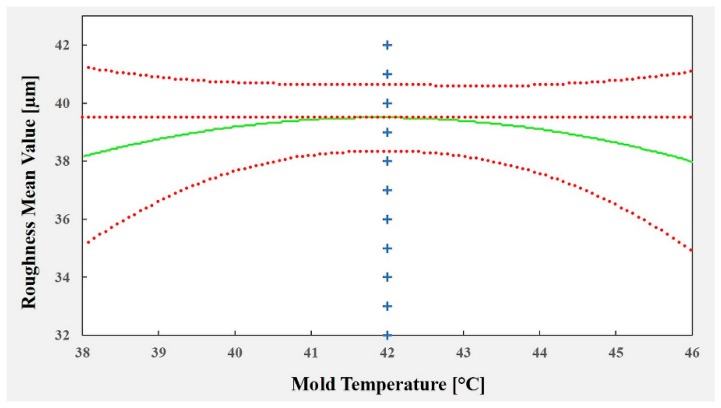
Effect of each predictor on the surface roughness model.

The plots shown in Figure 23 illustrate the effect of each predictor on the surface roughness model. To optimize the process parameters to replicate a texture (of 40 µm in roughness), which, as mentioned previously, is crucial for cell adhesion and growth (and reduced healing time), a roughness of 39.5 µm was chosen as a set point for the process. The corresponding values for each process predictor are: barrel temperature = 166 °C, injection pressure = 515 bar and mold temperature = 42 °C. Using these values, it is possible to predict a mean roughness of 39.49 µm in a 95% confidence interval between 38.36 µm and 40.63 µm.

## 4. Conclusions

The present paper has demonstrated the feasibility to replicate µ-components with high added value and advanced functionalities in a predictable and controllable manner. In particular, it has been shown that miniaturized biodegradable polymer, Purasorb PDLG 5010, can be overmolded onto a metal screw with high repeatability and good quality. Even with challenges such as a very thin overmolding layer, large surface area and textured thickness, orthopedic implants were manufactured successfully with tight tolerances.

The experimental trials with different parameter sets and replications showed that the process is stable, making it possible to obtain a process model based on the three selected predictors using the measured roughness as a quality mark. This proposed regression model was developed to understand the effects of the selected process parameters on the process. However, it was also used to optimize the values of the process parameters in order to have a process able to replicate effectively the desired overmolding layer composed of biodegradable polymer with the characteristics required for minimizing the healing phase.

The results showed that the overmolding mass value is not feasible as a quality mark. However, the optimum parameters were identified to obtain a predefined value of the surface roughness (Ra ≈ 40 µm) as a quantitative process response. This ultimately enables high quality overmolding of a metallic implant screw with a thin, micro-structured polymer layer. It is worth emphasizing that different processes were integrated into the injection molding process, i.e., the feeding and handling systems for automatic operation in order to eliminate human intervention and retain seamless manufacturing sequences.

As a step towards total quality in-line process control, the present study furthermore aimed at incorporating a fast 3D optical metrology system into the production line. Due to incompatibilities between the lighting and the polymer material, no measurements were possible without contaminating the screws to be used. Nevertheless, it was demonstrated that the proposed system gives a very good representation of the quality of the parts.

Further work will involve conducting a peel strength test to evaluate the adhesion force between the overmolding polymer layer and the metallic screw. This is to ensure that there will be sufficient adhesion force between both components (overmolding polymer layer and the metallic screw) that enables the polymer layer to remain in its position under actual working conditions. In addition, the implants overmolded with a PDLG (50/50 dl-lactide/glycolide copolymer) from Corbion Purac (Purasorb PDLG 5010) will be used for cell growth tests at a further stage in this study.

## Figures and Tables

**Table 1:** Requirements for the biodegradable polymer [31].

Glass transition temperature (°C)	>40
Elastic modulus (GPa)	>1
Degradation time (days)	28
Sterilizability	Yes
FDA approval	Yes

**Table 2:** Parameter set for each trial.

Trial (#)	Barrel Temperature (°C)	Injection Pressure (bar)	Mold Temperature (°C)	Parameter Set Index
1	160	500	42	1
2	164	452	40	2
3	164	452	44	3
4	164	548	40	4
5	164	548	44	5
6	170	420	42	6
7	170	580	42	7
8	170	500	38	8
9	170	500	46	9
10	170	500	42	15
11	170	500	42	15
12	170	500	42	15
13	170	500	42	15
14	170	500	42	15
15	170	500	42	15
16	170	500	42	15
17	170	500	42	15
18	176	452	40	10
19	176	452	44	11
20	176	548	40	12
21	176	548	44	13
22	180	500	42	14

**Table 3:** Analysis of variance (ANOVA) for obtainable roughness.

	SumSq	DF	MeanSq	F	*p*-Value
Barrel Temperature	6.0576	1	6.0576	17.416	0.0012919
Injection Pressure	2.3695	1	2.3695	6.8125	0.022798
Mold Temperature	0.15262	1	0.15262	0.43879	0.52023
Barrel Temperature:Injection Pressure	0.6217	1	0.6217	1.7874	0.20603
Barrel Temperature:Mold Temperature	4.5568	1	4.5568	13.101	0.0035174
Injection Pressure:Mold Temperature	16.268	1	16.268	46.772	1.8002 × 10^−5^
Barrel Temperature^2^	0.54577	1	0.54577	1.5691	0.23419
Injection Pressure^2^	16.258	1	16.258	46.743	1.8055 × 10^−5^
Mold Temperature^2^	3.5309	1	3.5309	10.152	0.0078309
Error	4.1738	12	0.34782		
